# Rodent models in Down syndrome research: impact and future opportunities

**DOI:** 10.1242/dmm.029728

**Published:** 2017-10-01

**Authors:** Yann Herault, Jean M. Delabar, Elizabeth M. C. Fisher, Victor L. J. Tybulewicz, Eugene Yu, Veronique Brault

**Affiliations:** 1 Institut de Génétique et de Biologie Moléculaire et Cellulaire, Illkirch, 1 rue Laurent Fries, 67404 Illkirch, France; 2 Centre National de la Recherche Scientifique, UMR7104, Illkirch, France; 3 Institut National de la Santé et de la Recherche Médicale, U964, Illkirch, France; 4 Université de Strasbourg, 67404 Illkirch, France; 5 T21 Research Society, Brain and Spine Institute (ICM), 75013 Paris; 6 Université Paris Diderot, Sorbonne Paris Cité, Unité de Biologie Fonctionnelle et Adaptative, UMR8251, CNRS, 75205 Paris, France; 7 INSERM U 1127, CNRS UMR 7225, Sorbonne Universités, UPMC Univ Paris 06 UMR S 1127, Institut du Cerveau et la Moelle épinière, ICM, 75013 Paris, France; 8 Brain and Spine Institute (ICM) CNRS UMR7225, INSERM UMRS 975, 75013 Paris, France; 9 Department of Neurodegenerative Disease, Institute of Neurology, University College London, London, WC1N 3BG, UK; 10 LonDownS Consortium, London, W1T 7NF UK; 11 The Francis Crick Institute, 1 Midland Road, London, NW1 1AT, UK; 12 Department of Medicine, Imperial College, London, SW7 2AZ, UK; 13 The Children's Guild Foundation Down Syndrome Research Program, Department of Cancer Genetics and Genetics Program, Roswell Park Cancer Institute, Buffalo, NY 14263, USA; 14 Department of Cellular and Molecular Biology, Roswell Park Division of Graduate School, Genetics, Genomics and Bioinformatics Program, State University of New York at Buffalo, Buffalo, NY 14263, USA

**Keywords:** Down syndrome, Mouse model, Chromosome engineering, Aneuploidy, Dosage-senstive gene

## Abstract

Down syndrome is caused by trisomy of chromosome 21. To date, a multiplicity of mouse models with Down-syndrome-related features has been developed to understand this complex human chromosomal disorder. These mouse models have been important for determining genotype-phenotype relationships and identification of dosage-sensitive genes involved in the pathophysiology of the condition, and in exploring the impact of the additional chromosome on the whole genome. Mouse models of Down syndrome have also been used to test therapeutic strategies. Here, we provide an overview of research in the last 15 years dedicated to the development and application of rodent models for Down syndrome. We also speculate on possible and probable future directions of research in this fast-moving field. As our understanding of the syndrome improves and genome engineering technologies evolve, it is necessary to coordinate efforts to make all Down syndrome models available to the community, to test therapeutics in models that replicate the whole trisomy and design new animal models to promote further discovery of potential therapeutic targets.

## Abstract (precis)

**Summary:** Mouse models have boosted therapeutic options for Down syndrome, and improved models are being developed to better understand the pathophysiology of this genetic condition.

## Introduction

Trisomy of human chromosome 21 (Hsa21; see Box 1 for a glossary of terms), which affects 1 in 700 live births globally (Canfield et al., 2006), gives rise to Down syndrome (DS), a condition that significantly impairs health and autonomy of affected individuals (Khoshnood et al., 2011; Parker et al., 2010). Despite the wide availability of prenatal diagnosis since the mid-1960s (Summers et al., 2007) and the introduction of maternal serum screening in 1984 (Inglis et al., 2012), the incidence of DS has not necessarily decreased (Natoli et al., 2012; Loane et al., 2013; de Graaf et al., 2016); in fact, prevalence is going up, largely because of increased lifespan and maternal age (which is the single biggest risk factor) (Sherman et al., 2007; Loane et al., 2013).

**Box 1. Glossary**

**Aneuploid:** having an abnormal or unbalanced number of chromosomes.

**Cardiac septation:** partitioning of the heart.

**Contextual and auditory-cue-conditioned fear task:** a test to study associative memory based on the association of environmental cues (the chamber for the context or a sound for the auditory cue) with an aversive stimulus (a light electric shock). The association of both stimuli will lead to a freezing, with almost no movement of the animal tested. Recording the percentage of immobility of the mouse after being placed backed in the environment or with the auditory cue 24 h after the shock gives an assessment of the associative memory.

**Euploid:** having a normal balanced number of chromosomes.

**Hypotonia:** a state of low muscle tone.

**Long-term potentiation (LTP):** an increase in synaptic response after high-frequency stimulation of neurons. A strategy used to test the plasticity and the consolidation of synapses.

**Morris water maze:** a test to study spatial memory, based on the normal behaviour of a mouse to exit a water maze using an external visual cue located outside a pool in which a small platform is hidden below the surface of the water. The path with the distance travelled and the time spent to reach the platform is indicative of function of spatial memory.

**Mosaic:** animal carries cells with different genotypes.

**Novel-object recognition**: a test to study non-spatial episodic memory based on the normal interest of the animal in exploring objects in its environment (an open field). The test evaluates recognition memory for previously explored objects by measuring the time spent sniffing a known versus a novel object.

**Positive thigmotaxis:** a behavioural preference displayed by some animals to be near or in close contact with the solid wall of an enclosure. The time spent in contact or close to the vertical wall of the open field is measured, in the exploration of a new environment.

**Transchromosomic**: a transgenic animal carrying a chromosome from a different species.

**Trisomy:** having a third copy of a given chromosome. Trisomy is associated with a number of human disorders, including Down syndrome.

**Ventriculomegaly:** enlargement or dilation of lateral brain ventricles.

**Y-maze:** a test involving a maze with three arms, which provides the animal with a choice: to visit the arm visited before or go to a new arm. This is a test that measures working memory.

A core set of features characterises most cases of DS, including specific cognitive disabilities, hypotonia (Box 1) at birth and characteristic craniofacial changes; however, other traits, such as cardiac defects and susceptibility to leukemias, affect only a subset of individuals with DS (OMIM 190685; ORPHA870). Later in life, the majority of DS individuals will develop Alzheimer's disease (AD; approximately 60% by the age of 65), making trisomy 21 the most common genetic cause of this neurodegenerative disease (Ballard et al., 2016; Dekker et al., 2015; Head et al., 2015; Wiseman et al., 2015).

The phenotypes observed in DS are likely to arise because of dosage sensitivity of Hsa21 genes and associated gene-environment interactions (Antonarakis et al., 2004; Antonarakis, 2016; Beach et al., 2017), and/or a global effect of the extra chromosome on chromatin regulation and methylation (Letourneau et al., 2014; Hervé et al., 2016; Mendioroz et al., 2015). Studies of patients carrying rare segmental duplications of Hsa21 subregions have highlighted the role of specific chromosomal regions in DS pathophysiology (Korbel et al., 2009; Korenberg et al., 1994; Lyle et al., 2009; Delabar et al., 1993). In addition, studies using animal models have confirmed the involvement of homologous regions and shown how some regions with orthologues of individual Hsa21 dosage-sensitive genes are key for DS features (discussed in detail below). A few genes not located on Hsa21 have been shown to contribute to individual phenotypic variation (Roper and Reeves, 2006). Analysis of individuals with a segmental duplication of Hsa21 has been key to building up a phenotypic map and defining a critical DS region (Delabar et al., 1993; Lyle et al., 2009; Korbel et al., 2009; Korenberg et al., 1994; Rahmani et al., 1989). Nevertheless, with only about 60 duplications reported so far in the literature, the resolution of this map is very low. Moreover, duplications that do not induce strong phenotypes, or that lead to embryonic death, are not represented in published studies (Rovelet-Lecrux et al., 2006). A more detailed understanding of the DS genotype-phenotype relationship in humans would require a systematic analysis of very large numbers of individuals and of stillborns. Indeed, 31-54% of DS pregnancies lead to spontaneous foetal loss (Loane et al., 2013; Morris et al., 1999; Morris and Wald, 2007).

This Review focuses on the use of rodent models of DS, which have been essential for the determination of genotype-phenotype relationships for this syndrome. Owing to the genetic tractability of this animal, the most useful DS models to date have been derived from the laboratory mouse. Mice are highly amenable to genome engineering, including through chromosome engineering, to generate precisely defined large genomic segmental duplications to model chromosomal disorders (Brault et al., 2006; Yu and Bradley, 2001; Ramirez-Solis et al., 1995; Hérault et al., 1998; Tybulewicz and Fisher, 2006). Mouse models have also provided platforms for testing interactions between cell and tissue types, responses in the organism, and candidate therapeutics for DS. We highlight the approaches and technologies that have been used to generate mouse models of DS in recent years, and also discuss how the study of these models has brought new knowledge about DS pathophysiology, including key candidate pathways and genes, as well as providing new therapeutic approaches.

## Building up a compendium of DS models

The rapid development of genetic engineering in recent years has stimulated the generation of multiple DS mouse models. A variety of transgenic models for candidate genes were developed in early attempts at modelling DS in mice (Dierssen et al., 2009), and we discuss such experiments briefly in a later section. Here, we discuss DS mouse models that contain larger trisomic or duplicated chromosomal segments, thereby mimicking the trisomy observed in humans.

### Early mouse models of trisomy 21

**Fig. 1.**
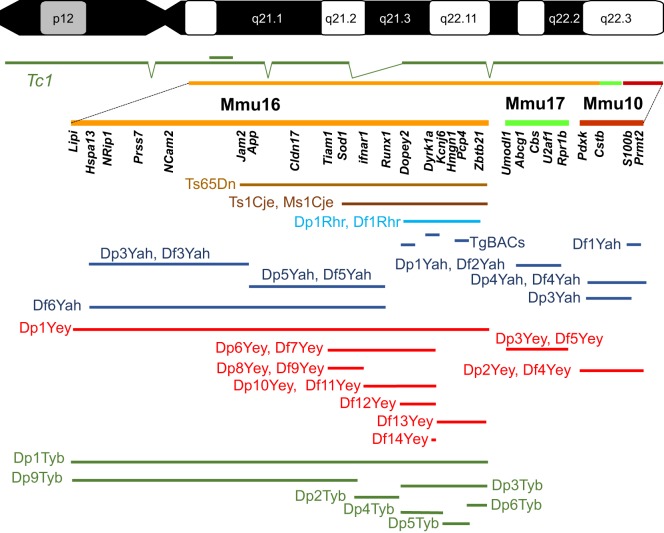
**Mouse models of DS.** Human chromosome 21 (p and q arms; G-banding) is depicted at the top of the figure, with the mouse genome orthologous region found on chromosome 16 (Mmu16), Mmu10 and Mmu17 shown respectively in orange, light green and red. A few known genes that are homologous to Hsa21 genes in the DS critical region are listed below each chromosome. The transchromosomic Tc1 mouse model is shown in dark green, with deletions and a duplication (double bar) relative to Hsa21 depicted. Below, the segment of the DS critical region encompassed in different mouse models for DS is illustrated. The original Ts65Dn (Reeves et al., 1995) and Ts1Cje (Sago et al., 1998) models (shown in brown) originated by accidental translocation of Mmu16 segments respectively on Mmu17 and Mmu12, with some additional changes (Duchon et al., 2011b; Reinholdt et al., 2011). Olson et al. (2004) published the first engineered duplication (Dp) and deletion [deficiency (Df)] for the DS critical region (light blue). New models have been developed in the last 10 years by the authors of this Review, as shown in dark blue (Duchon et al., 2011a; Lopes Pereira et al., 2009; Besson et al., 2007; Marechal et al., 2015; Sahun et al., 2014; Raveau et al., 2012; Arbogast et al., 2015; Brault et al., 2015b), red (Jiang et al., 2015; Liu et al., 2011, 2014; Yu et al., 2010a,b,c; Li et al., 2007) and green (Lana-Elola et al., 2016). TgBACs, a few models for BAC or PAC (P1-derived artificial chromosome) transgenic lines.

Over the approximately 75-million years that separate humans and mice in evolutionary time, the chromosomes have rearranged such that Hsa21 has three orthologous regions on mouse chromosomes 10, 16, 17 in which gene order and orientation are conserved (Mmu10, 16, 17; Mmu for *Mus musculus*; Fig. 1). Hsa21 carries 222 protein-coding genes, including 49 that encode keratin-associated proteins and are clustered on Hsa21q, and 325 non-protein-coding genes (Gupta et al., 2016). Of the 158 mouse genes that are homologous to human protein-coding genes, most of them lie on Mmu16 (a total of 102) between *Lipi* and *Zbtb21*, a few on Mmu17 (19) between *Abcg1* and *Rrp1b*, and the rest on Mmu10 (37) between *Pdxk* and *Prmt2*. Of the non-coding genes, 75 elements, such as those encoding miRNAs, are well conserved and are distributed across all three mouse chromosomes (Gupta et al., 2016). Because the equivalent genetic elements are distributed between regions on three different chromosomes, modelling trisomy 21 in the mouse is not straightforward (Antonarakis et al., 2004). In addition, a handful of human genes [such as the prostate-, ovary-, testis- and placenta-expressed ankyrin domain family member D (*POTED*)] are not conserved in the mouse, and there are mouse genes, such as integrin beta-2-like (*Itgb2l*) located between *Igsf5* and *Pcp4* in the Hsa21 homologous regions, that have no human homologues. Furthermore, we do not yet have a clear picture of the role and function (if any) of many pseudogenes (Gupta et al., 2016).

Early modelling was attempted by studying mice with full trisomy of mouse 16 (Gropp et al., 1975; Gropp, 1974). These animals have numerous defects, including, for example, cardiac septation deficits (Box 1) (Webb et al., 1999); however, they do not model DS because the majority of genes that are triplicated in this model are from regions of Mmu16 without homology to Hsa21. Furthermore, these animals die at birth and so cannot give insight into processes beyond this stage.

The field of DS investigation moved forward by the discovery in 1990 and the phenotypic description in 1995 of the Ts65Dn mouse (Fig. 1) (Reeves et al., 1995; Davisson et al., 1990). This mouse has a translocation that results in an extra-small chromosome made up of a fusion of the *App-Zbtb21* region orthologous to Hsa21 found on Mmu16 with the centromeric region of Mmu17; thus, the mouse shows aneuploidy (Box 1). The extra region of Mmu16 includes 90 conserved protein-coding Hsa21 gene orthologues (Choong et al., 2015; Gupta et al., 2016). The Ts65Dn mouse was the main model used to study DS for at least two decades and has provided many new insights (see below). However, the animal carries three copies of an extra segment (arising from Mmu17) with non-DS-related genes, including ∼35 protein-coding genes, 15 non-protein-coding genes and 10 pseudogenes (Duchon et al., 2011b; Reinholdt et al., 2011). Moreover, even though some Ts65Dn males are fertile (Moore et al., 2010), transmission is usually achieved through the maternal germline. This might affect the phenotype of the trisomic progeny and their disomic littermates because the mothers are trisomic, generally unlike the situation in humans.

Other models of partial trisomy 16, the Ts1Cje (trisomic for the *Sod1*-*Zbtb21* region, shown in Fig. 1) and the Ts2Cje (harbours a Robertsonian translocation between the extra chromosome in Ts65Dn and mouse chromosome 12) (Fig. 1, Table 1), have made important contributions to our understanding of DS. Nevertheless, as with Ts65Dn, they were generated by chance rather than design, and carry additional genetic modifications that could have an impact on phenotypes (Sago et al., 1998; Villar et al., 2005).

**Table 1.**
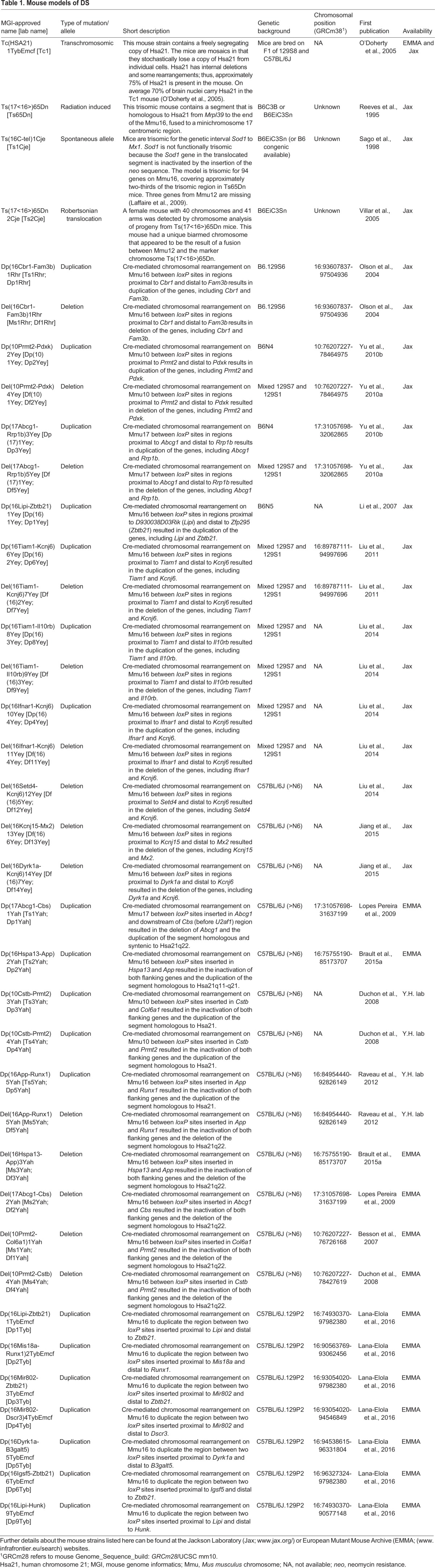
**Mouse models of DS**

### Advances in engineering DS mouse models

The field of DS modelling in mice changed significantly in the mid-2000s with the advent of two new types of mice: one that is transchromosomic (Box 1) and those that are chromosome engineered.

In 2005, V.L.J.T., E.M.C.F. and colleagues published the first transchromosomic mouse line (O'Doherty et al., 2005), namely Tc1 [formally called Tc(Hsa21)1TybEmcf]. The line was generated using irradiation microcell-mediated chromosome transfer into embryonic stem (ES) cells, leading to a freely segregating copy of Hsa21, transmitted through the germline. In Tc1, human Hsa21 sequences are expressed in the mouse at the mRNA, protein and functional levels (Ahmed et al., 2013; Reynolds et al., 2010; O'Doherty et al., 2005). For example, targeting the overexpressed transcripts encoded by four genes restored VEGF-dependent normal angiogenic responses in Tc1 mice (Reynolds et al., 2010). However, the human chromosome is lost stochastically from cells, and so the resulting mice are mosaics (Box 1): some cells carry the supernumerary Hsa21, whereas others do not. Also, complete sequencing of the human chromosome (Gribble et al., 2013) has revealed that it was rearranged in the process, probably due to γ-ray-induced *de novo* rearrangements, leading to incomplete trisomy. Nevertheless, this mouse remains a unique complement to the models that help us to understand DS and has given insight into the condition (Hall et al., 2016; Peiris et al., 2016; Powell et al., 2016; Witton et al., 2015).

**Fig. 2.**
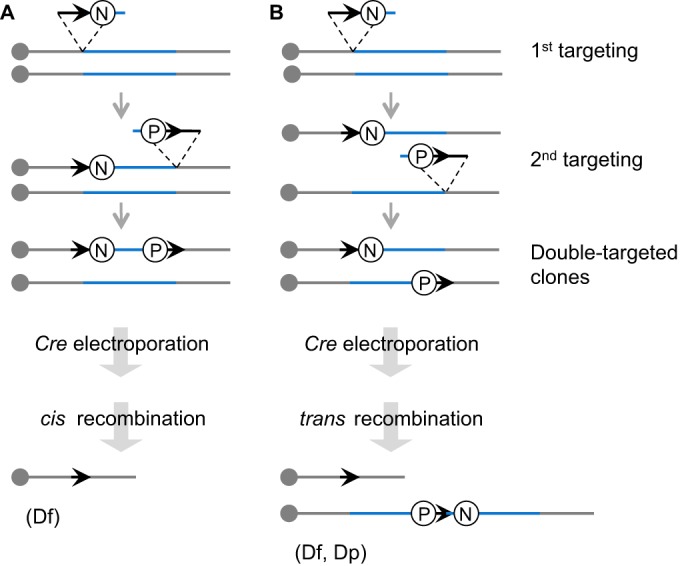
**Cre-*loxP*-mediated chromosomal engineering in mice.** A *loxP* site (arrow) is targeted into the first endpoint of the engineered segment (blue) in the embryonic stem (ES) cell genome with a positive selectable marker, such as *neo* (the neomycin-resistance gene; N). Next, a second *loxP* site is targeted to the other endpoint with another positive selectable marker such as *puro*, the puromycin resistance gene (P). A Cre expression vector is then transferred by electroporation into double-targeted ES cell clones. If two *loxP* sites are targeted onto the same chromosome homologue and oriented in the same direction (*cis*), recombination between the sites will lead to a deletion (Df; A). If two *loxP* sites are targeted onto two separate homologues and oriented in the same direction (*trans*), the recombination will lead to a duplication (Dp) and the reciprocal deletion (Df) (B).

In the mid-1990s, an approach to generate precise chromosomal rearrangements, including duplications and deletions, was developed in mice (Ramirez-Solis et al., 1995). This technology has radically expanded the available mouse resources for understanding DS by facilitating the design of partial trisomies (Yu and Bradley, 2001; Olson et al., 2004) and producing the most complete model we have: the triple trisomic mouse. This mouse is partially trisomic for the Mmu10, 16 and 17 regions that are homologous to Hsa21 (Yu et al., 2010b). Briefly, the desired chromosomal rearrangement is first engineered in mouse ES cells with two steps of gene targeting to introduce *loxP* sites upstream and downstream of the region of interest with selectable markers, and one additional step leading to the reconstruction of a selectable minigene after Cre expression (Fig. 2). A specific orientation of *loxP* targeted on a chromosome must be achieved by appropriate design to induce the chromosomal change. Identification of ES cell clones harbouring an engineered chromosomal rearrangement is facilitated by positive selection of expression of the minigene. Chromosomal duplications and deletions can be precisely verified by Southern blot analysis, fluorescence *in situ* hybridisation, array-based comparative genome hybridisation or whole-genome sequencing (Yu et al., 2010b; Gribble et al., 2013). Selected ES cells are used to establish the corresponding mouse line. Alternatively, recombination between *loxP* sites can be achieved *in vivo* (Brault et al., 2007; Hérault et al., 1998) in a mouse carrying the two *loxP* sites and one specific Cre driver expressed in the germline. In this case, no additional construct is needed at the frontier of the recombined fragments, minimising potential interference associated with the reconstruction of a minigene. Altogether, these Cre/*loxP*-based technologies, carried out independently in two laboratories, resulted in the generation of several mouse models with segmental duplications encompassing different segments of the mouse chromosomes orthologous to Hsa21 (Hérault et al., 2012).

The crucial step needed for inserting *loxP* sites is now facilitated using recombinant transposon-mediated insertion and further selection (Chen et al., 2013; Ruf et al., 2011). In addition, *loxP* site insertions in the mouse genome generated by transposition have been captured in the TRACER resource with precise location and orientation (http://tracerdatabase.embl.de), reducing the time needed to generate new DS models to 3 years (Chen et al., 2013).

However, a major revolution is now underway with the development of an even faster method – CRISPR-mediated rearrangement (CRISMERE) – which is based on CRISPR/Cas9 genome-editing technology (Birling et al., 2017). Two pairs of small guide RNA (sgRNA), each pair selected either upstream or downstream of the region of interest, are injected with the Cas9 nuclease into one-cell mouse embryos that are reimplanted. Newborns are analysed for chromosomal modifications and are bred to select carrier individuals with the new rearrangement in the next generation. The making of *in vivo* duplications, deletions and inversions of genomic segments of up to 34 Mb using CRISMERE requires less time than it takes to observe germline transmission of recombined ES cells. CRISMERE is not limited to the mouse and has been successfully used in rats (Table 1) (Birling et al., 2017) and can be applied to primates. In the rat (*Rattus norvegicus*) genome, the Hsa21 homologous regions are located on two chromosomes, Rno11 and Rno20. On Rno11, the *Lipi-Zbtb21* segment is almost identical to the homologous region located on the Mmu16, whereas Rno20 harbours a unique segment for the *Umodl1*-*Prmt2* interval (Fig. 1). Using CRISMERE, new models encompassing both regions have been generated (Birling et al., 2017). These models have the potential to facilitate testing of therapies in both mouse and rat models to enable stronger validation prior to assessment in clinical trials.

Nowadays, the development of DS models is no longer limited at the technical level but rather more at the conceptual level, i.e. in terms of the challenges associated with precise delineation of the region of interest for a particular phenotype. A key question is whether it is better to define smaller regions of interest and generate more models or make models for larger segments to recapitulate the human trisomy. In any case, enhancing our understanding of the link between phenotype and genotype is critical, as discussed below.

## Assessing the genotype-phenotype relationship in DS

Many DS features show variable penetrance (http://omim.org/entry/190685#clinicalFeatures) (Kruszka et al., 2017; Roizen and Patterson, 2003). Understanding the molecular basis of this huge variability between individuals could inform the development of therapies to modulate specific features of the syndrome. Variability in DS includes the degree of learning difficulties observed – in 39.4% of cases, IQ typically ranges between 50 and 70, but 1% of affected individuals have an IQ around the borderline function range of 70-80 (Antonarakis et al., 2004). The presence of cardiac anomalies, and the incidence of leukaemia, autoimmune diseases, AD pathology and dementia, as well as accelerated ageing, are also quite variable (Antonarakis et al., 2004). In order to better understand the physiopathology of the disease and to correlate genotype with the features observed in patients, studies in animal models and particularly mouse models have been critical (Gupta et al., 2016).

One of the first consequences of DS is the alteration of embryonic development, leading at the extreme to gestational loss in humans (Loane et al., 2013; Morris et al., 1999; Morris and Wald, 2007). This phenotype is also observed in Tc1 mice, in Dp1Yey and Dp1Tyb mice (generated in two independent groups), which duplicate the region from *Lipi* to *Zbtb21* (Fig. 1), in the Ts65Dn mouse, and to some extent in the Ts1Cje mouse (Arbogast et al., 2015; Raveau et al., 2012; Yu et al., 2010b; Li et al., 2007; Guedj et al., 2016; Lana-Elola et al., 2016; O'Doherty et al., 2005). Reducing the dosage of the 7.7 Mb *App-Runx1* region, containing 54 protein-coding genes and 25 keratin genes, in Ts65Dn mice rescued impaired postnatal viability, and deletion of this region resulted in severe phenotypes and lowered viability, suggesting the presence of critical genes in the interval (Arbogast et al., 2015; Raveau et al., 2012).

In light of the characteristic intellectual deficiency in human DS, many types of learning and memory have been monitored in DS mouse models to explore which part of the brain is affected by the trisomy (Das and Reeves, 2011; Gupta et al., 2016; Xing et al., 2016; Belichenko et al., 2015; Jiang et al., 2015; Zhang et al., 2014; Arbogast et al., 2015; Brault et al., 2015b; Marechal et al., 2015; Sahun et al., 2014; Hérault et al., 2012). The open-field (OF) test (Stanford, 2007) has been used in these studies to assay locomotor activity, exploration and anxiety. Episodic memory (involving the perirhinal cortex and the hippocampus) was a particular focus, with tests for non-spatial learning such as the novel-object recognition (NOR; Box 1) applied with two retention times: 1 h or 24 h (Cohen and Stackman, 2015). Short-term working memory was assessed with continuous spontaneous alternation behaviour mostly using the Y-maze test (Box 1) (Hughes, 2004). Spatial memory, which involves several regions of the brain (hippocampus, striatum, basal forebrain, cerebellum and cerebral cortex), has been explored in DS mice using the Morris water maze (MWM; Box 1) test (D'Hooge and De Deyn, 2001; Morris, 1984). For associative memory, the contextual and auditory-cue-conditioned fear task [FC (fear conditioning); Box 1] (Paylor et al., 1994; Mátyás et al., 2014; Lee et al., 2011) was used to test the connection between the hippocampus, frontal cortex, cingulate cortex and amygdala and the mediodorsal thalamic nucleus. These studies point to an important role for *DYRK1A* (discussed in the section below). *DYRK1A*, the mammalian orthologue of *Drosophila minibrain kinase (mnb)* (Tejedor et al., 1995), encodes a proline/arginine-directed dual-specificity kinase, and is overexpressed both in the brain of trisomic mice and of individuals with DS (Dowjat et al., 2007). Three copies of *Dyrk1a* are necessary and sufficient to induce several deficits in NOR (at 1 and 24 h) and FC, but only result in delayed learning in the MWM (García-Cerro et al., 2014; Pons-Espinal et al., 2013; Dierssen and de Lagrán, 2006; Altafaj et al., 2001; Duchon and Herault, 2016). Interestingly, the Ts1Rhr trisomy mouse, which is trisomic for the DS critical region with 33 genes including *Dyrk1a*, displayed deficits in the OF test and in NOR, with 24 h of retention (Belichenko et al., 2009); additionally, trisomy of this region was necessary to alter spatial memory in Ts65Dn mice (Olson et al., 2007). In Ts65Dn, Ts1Cje and Ts1Rhr mice, long-term potentiation (LTP; Box 1), a measure of synaptic plasticity, could be induced only after blocking GABA(A)-dependent inhibitory neurotransmission in the fascia dentata, a structure that receives inputs from the perirhinal cortex (Belichenko et al., 2009; Kleschevnikov et al., 2012b); this result is indicative of excessive neuronal inhibition and is consistent with previous observations of Ts65Dn mice (Kleschevnikov et al., 2012b; Fernandez et al., 2007). In addition, widespread enlargement of dendritic spines and decreased density of spines in the fascia dentata were observed, which could explain the overall reduced activation of neuronal activity (Belichenko et al., 2009; Haas et al., 2013). Thus, cognitive impairment in DS seems to derive from molecular and structural changes related to an altered copy number within this 33-gene region. This conclusion was confirmed when combining Dp1Yey mice either with deletion of the *Std4-KcnJ6* interval or *Kcnj15-Mx2*, which showed that both regions contain dosage-sensitive genes contributing to cognitive phenotypes (Jiang et al., 2015).

The Ts65Dn mouse model also displays lower performance in finding a hidden platform compared to controls in the MWM task at 4 months of age (Reeves et al., 1995; Netzer et al., 2010; Olmos-Serrano et al., 2016b), but this phenotype is not consistently observed in 2- to 4-month-old Dp1Yey mice (Yu et al., 2010c; Goodliffe et al., 2016). Nevertheless, learning is impaired for both models in a variant of the MWM test at the age of 2-3 months (Goodliffe et al., 2016; Olmos-Serrano et al., 2016a). Overall, the results obtained from these studies are difficult to compare owing to differences in age of tested individuals and more importantly in the protocols or the genetic background used. Thus, there is a strong need to better standardise experimental protocols to allow for more equivalent cross-laboratory comparisons.

Another important point is that combining models with different segmental trisomies can alter the phenotypic outcomes (Jiang et al., 2015; Duchon et al., 2011a; García-Cerro et al., 2014; Salehi et al., 2006). Studies in such mice strengthen the evidence for the multigenic nature of DS, already pointed to in human genetic studies (Korbel et al., 2009; Korenberg et al., 1994; Lyle et al., 2009), with multiple genes interacting to induce the frequently observed intellectual disability that characterises DS. One of the main conclusions is that the hippocampus is a key hub whose dysfunction is observed in many DS mouse models, altering many types of memory, including, for example, the function of the place cells, a type of hippocampal pyramidal neuron that acts to define a cognitive map needed for spatial memory (Witton et al., 2015).

DS universally causes the typical plaques and tangles of AD to appear in the brain by the age of 40, and current figures show that two-thirds of people with DS develop dementia by the age of 60 (Wiseman et al., 2015). Individuals with DS develop Alzheimer's-like pathologies comparatively early in life, including progressive degeneration of basal forebrain cholinergic neurons (BFCNs). The Ts65Dn mouse model exhibits elevated levels of β-amyloid (Aβ) peptide, as well as atrophy of BFCNs. Although the mechanisms are not yet fully understood, the appearance of the pathology almost certainly arises from overexpression of the Hsa21 gene *APP*, which is known to cause early-onset AD when present in three copies, as shown in very rare families with small internal chromosomal duplications that include this gene (Rovelet-Lecrux et al., 2007, 2006). In line with this hypothesis, *App* triplication is necessary for the age-dependent BFCN loss observed in the Ts65Dn mouse (Salehi et al., 2006; Granholm et al., 2000) and for neuronal abnormalities in the endosomal compartment, also reported in these mice (Cataldo et al., 2008, 2000).

Intriguingly, not everyone with DS develops dementia, although all individuals with DS over the age of 40 show evidence of amyloid plaques. Individuals with duplicated *APP* have dementia onset that is fully penetrant, between 39 to 64 years of age (Wiseman et al., 2015). Conversely, individuals with DS (and thus *APP* in triplicate) show a wide range in age-of-onset of dementia, and can live into their 70s with no sign of dementia (Karmiloff-Smith et al., 2016; Krinsky-McHale et al., 2008; Ghezzo et al., 2014). Thus, it seems likely that there are also protective factors for AD on Hsa21, and these might be important for understanding and treating dementia in the euploid (Box 1) population. Although the genes involved in modulating AD phenotypes remain to be determined, crosses of different types of AD and DS mouse models to humanised APP will give insight into molecular processes (Choong et al., 2015; Hamlett et al., 2016).

Insights into congenital heart defects (CHDs) in DS have also been gained using mouse models. In humans, 40% of DS newborns present with a CHD (Antonarakis et al., 2004). In mice, CHDs were observed during development in Tc1 (O'Doherty et al., 2005), Ts65Dn (Moore, 2006), Dp1Yey (Li et al., 2007), Dp1Tyb (Lana-Elola et al., 2016) and Ts1Cje (Guedj et al., 2016) embryos. Detailed investigation with several trisomic models refined a critical interval between *Ifnar1* and *KcnJ6* (Fig. 1) (Raveau et al., 2012; Liu et al., 2011) and a role for the most distal part of Mmu16 from *mir802* to *Zbtb21* (Lana-Elola et al., 2016). In addition, overexpression of *Jam2*, a gene located upstream of *App* that encodes junctional adhesion molecule 2 found in the tight junctions of endothelial cells, modifies the activity of the matricellular protein cysteine-rich with EGF-like domain protein 1 (CRELD1), leading to enhanced septal defects in Ts65Dn mice (Li et al., 2016). Reducing the dosage of another key gene for heart development located outside of Hsa21 homologous regions, T-box transcription factor *Tbx5* worsens CHDs in the Ts65Dn mouse model, evidenced by increasing aortic and atrial-ventricular septal defects (Polk et al., 2015). These studies indicate that several Hsa21 homologous regions contribute to CHDs in DS and that additional genes involved in normal heart development can modify the severity of the heart phenotypes observed in DS models.

Additional DS-related features, such as craniofacial changes, have been described in mouse models, and certain trisomic genes or regions have been implicated in these phenotypes (Starbuck et al., 2014; Richtsmeier et al., 2002, 2000). A mouse-based study has also shed light on hypotonia, a major phenotype observed in DS newborns (Vicari, 2006). Analysis of a mouse model carrying three copies of the *Hspa13-App* region (Fig. 1) shows changes in locomotor activity and in muscle strength and physiology, suggesting the contribution of muscular deficits in the periphery (Brault et al., 2015a).

This summary is not exhaustive and many additional DS features have been explored using mouse models, such as the mineralisation of long bones (Blazek et al., 2015), the risk of chronic otitis media (Bhutta et al., 2013), and the higher risk of developing myeloproliferative disorders and cancer (Ng et al., 2015; Sussan et al., 2008; Malinge et al., 2012; Alford et al., 2010; Yang et al., 2016). Each mouse strain has the potential to provide unique information, and a range of animals, aneuploid and partially trisomic, are important to pinpoint specific candidate genes and dissect the underlying molecular mechanisms.

## Identification of molecular mechanisms and candidate genes for DS cognitive features

The analysis of DS mouse models has facilitated the identification of specific Hsa21 genes involved in DS features. Initially, DS candidate genes were pinpointed by human genetic analyses or by parallel knowledge of gene function (Antonarakis, 2016; Antonarakis et al., 2004). Two basic experimental approaches in mice have been applied either to increase or decrease the expression of a candidate gene. As described above, a 33-gene region has been identified as being crucial for cognitive impairment in DS, based on a number of mouse behavioural studies (Belichenko et al., 2009; Olson et al., 2007). Among the genes from this region, *Dyrk1a* was an attractive candidate for inducing cognitive-impairment phenotypes.

Large genomic fragments such as yeast artificial chromosomes (YACs) containing mouse DNA of the locus (Smith and Rubin, 1997; Smith et al., 1995) or bacterial artificial chromosome (BAC) constructs covering the human (Ahn et al., 2006) or the mouse (Guedj et al., 2012) gene, were developed in order to express *Dyrk1a* with a pattern of expression similar to the endogenous gene. The YAC transgenic mouse was used to demonstrate the role of *Dyrk1a* in DS cognitive impairment (Sebrié et al., 2008; Rachidi et al., 2007; Roubertoux et al., 2006; Branchi et al., 2004). The evidence for this was reinforced by applying transgenic approaches to overexpress *Dyrk1a* alone in mice either by using expression vectors driven by an exogenous promoter (Altafaj et al., 2001, 2013; Grau et al., 2014; Martinez de Lagrán et al., 2004) or by using BAC encoding human or mouse *DYRK1A* (Ahn et al., 2006; Guedj et al., 2012). All lines played an important role in understanding the molecular consequences induced by *DYRK1A* overdosage and provided important support for demonstrating molecular alterations in synaptic plasticity pathways, particularly expression changes in GABAergic- and glutamatergic-related proteins (Ahn et al., 2006; Park et al., 2012, 2010; Song et al., 2015, 2013; Souchet et al., 2015; Rachdi et al., 2014; Laguna et al., 2013; Guedj et al., 2012; Souchet et al., 2014). Similar alterations were observed in models with partial trisomy of Mmu16, Ts65Dn and Dp(16)1Yey, and were reversed in the *Dyrk1a*^+/−^ model (Souchet et al., 2014). Overexpression of *Dyrk1a* also decreased firing rate and γ-frequency power in the prefrontal network of anesthetised and awake mice, indicating that excess levels of this gene reinforce neuronal inhibition (Ruiz-Mejias et al., 2016).

Reducing the *Dyrk1a* dosage in Ts65Dn mice by crossing Ts65Dn females with heterozygous *Dyrk1a^+/−^* male mice revealed that normalization of the *Dyrk1a* copy number improves spatial working, reference memory and contextual conditioning, as well as rescuing hippocampal LTP (García-Cerro et al., 2014). Similar results were obtained by crossing Dp1Yey mice with *Dyrk1a*-knockout mice: rescued trisomic mice with only two functional copies of *Dyrk1a* showed a better performance in the T-maze and FC assays (Jiang et al., 2015). Concomitant with these functional improvements, normalisation of the *Dyrk1a* expression level in trisomic mice restored the proliferation and differentiation of hippocampal cells in the adult dentate gyrus (DG), and the density of GABAergic- and glutamatergic-synapse markers in the molecular layer of the hippocampus (García-Cerro et al., 2014).

Additional genes have been implicated in DS phenotypes in mice. *App* triplication was shown to impact BFCN degeneration, consistent with a role for this AD-associated gene in DS impaired cognition (Salehi et al., 2006). Regulator of calcineurin 1 (Rcan1) inhibits the calcineurin-dependent signalling pathway and its aggregation is controlled by Dyrk1a phosphorylation (Song et al., 2013). Increasing *RCAN1* gene dosage impairs hippocampal LTP (Xing et al., 2013), whereas genetic rescue experiments restore sympathetic nervous system development in Dp1Yey mice (Patel et al., 2015). The gene encoding calmodulin regulator protein Purkinje cell protein 4 (*Pcp4*) has been overexpressed using a P1-phage vector (PAC) to generate transgenic mice that display cerebellar defects (Mouton-Liger et al., 2014, 2011). Recently, overexpression of this gene was implicated in the brain ventriculomegaly (Box 1) observed in Ts1Cje mice and in DS-affected humans, and it is thought that the mechanism involves impaired cilia function in ependymal cells, which form the lining of the brain ventricular system (Raveau et al., 2017). Another gene that has been linked to DS is *Kcnj6*, which encodes potassium-voltage-gated channel subfamily J member 6. This gene has been shown to contribute to CHDs (Lignon et al., 2008) and to cognitive defects, together with another gene not yet identified (Jiang et al., 2015; Joshi et al., 2016; Cooper et al., 2012). The gene encoding cystathionine β-synthase (*Cbs*), which is involved in the methionine/cysteine cycle, is overexpressed in the DS brain (Ichinohe et al., 2005). Comparable overexpression of *Cbs* using a PAC transgenic line leads to changes in behaviour and LTP in mouse (Régnier et al., 2012), with similar phenotypes being observed in trisomic mice involving larger segments that include *Cbs* (Lopes Pereira et al., 2009; Yu et al., 2010c).

Several pathways that are perturbed in DS mouse models have been brought to light using transcriptomic and proteomic approaches. A meta-analysis of DS data, selected from human and mouse studies, unveiled perturbed neurological processes involved in neurodegeneration, axon guidance and nerve growth factor (NGF) signalling (Vilardell et al., 2011). A few Hsa21 genes (*SOD1*, *APP*, *DONSON*, *TIAM1*, *COL6A2*, *ITSN1* and *BACE2*) and the brain-derived neurotropic factor (BDNF)-dependent pathway, involved in growth, differentiation and survival of neurons, were found to be altered. An elevated level of BDNF and of Akt-mTOR/Ras-ERK signalling was observed in the hippocampus of Ts1Cje mice, and normal mTOR activity could be restored by treatment with the mTOR inhibitor rapamycin (Troca-Marín et al., 2014, 2011). In agreement, Ahmed et al. (2013) also showed that mTOR signalling is deregulated in Tc1 mouse brains.

There are likely to be multiple signalling pathways affected in the DS brain, however. In a recent systems biology study, the transcriptomes of cells taken from DS-affected human fetuses (and unaffected controls) were compared to transcriptome data from the embryonic forebrains of three mouse models (Dp1Yey, Ts65Dn and Ts1Cje) (Guedj et al., 2016). Their analyses revealed that a large panel of cellular processes (cellular stress response, DNA-repair signalling, regulation of cell cycle checkpoints, kinetochore organisation, proteolytic activity and anti-apoptotic genes) and molecular pathways (neurogenesis and neuronal differentiation, mitochondrial function, oxidative stress response, and inflammation) is dysregulated in DS mouse models and humans, indicating that the disease mechanisms are likely to be similar in both species. Another study, involving multi-regional transcriptome analysis of human DS and euploid foetal brains, pointed to misregulation of genes involved in the differentiation of oligodendrocytes and in myelination (Olmos-Serrano et al., 2016a). These findings were confirmed by analysis of Ts65Dn trisomy mice in the same study, highlighting that defects in white matter function could play a part in DS physiopathology. Lastly, epigenetic profiling has revealed multiple loci with altered CpG methylation in human DS and in mouse models, and this phenomenon may reflect increased dosage of Hsa21-linked methylation-pathway genes, such as *DNMT3L*, *SLC19A1* and others, as well as overexpression of key Hsa21-linked transcription factors, such as RUNX1, which could affect epigenetic patterns where they bind DNA (reviewed in Do et al., 2017). Collectively, these studies give an idea of the complexity of DS and emphasise the need to use an integrative approach that includes human samples and animal models, analysed at different periods in development (for example, foetal, early postnatal, young and late adult stages), to better understand the sequence of altered cellular processes and affected pathways in this disease.

## Therapeutic proof-of-concept for DS

In the last 10 years, a number of studies have sought to assess the efficacy of candidate therapeutic interventions for DS using mouse models. A summary of studies that have explored strategies for rectifying molecular, cellular or systemic defects in DS using mice is given in Table 2.

**Table 2.**
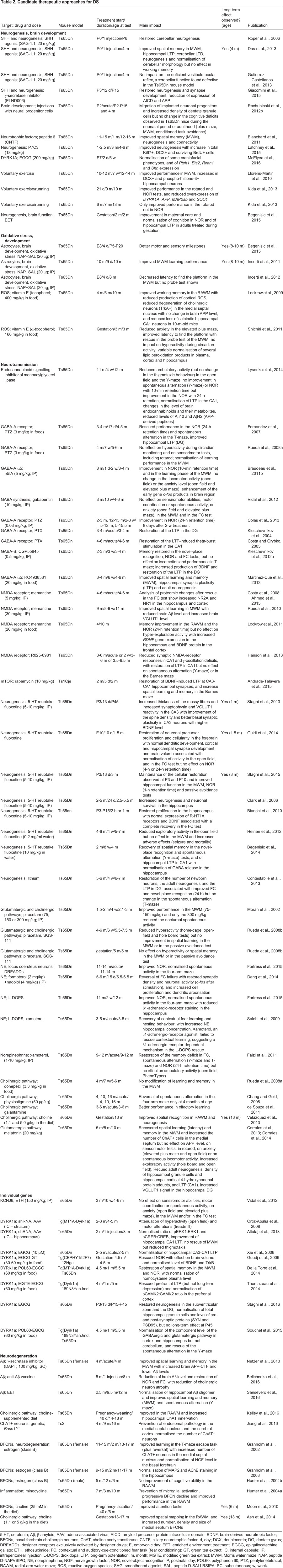
**Candidate therapeutic approaches for DS**

Some studies have attempted to target defects in neurogenesis and brain development in DS mouse models. The origin of the granule cell deficit in Ts65Dn has been traced to precursors in early postnatal development, which show a substantially reduced mitogenic response to hedgehog protein signaling (Roper et al., 2006; Baxter et al., 2000), a crucial pathway in development. Activation of the sonic hedgehog (SHH) pathway can be achieved by intraperitoneal injection of smoothened agonist (SAG) at postnatal day 2 (P2). Treated Ts65Dn pups have partially corrected cerebellar neurogenesis and hippocampal LTP defects, resulting in improved spatial memory (Das et al., 2013; Gutierrez-Castellanos et al., 2013; Roper et al., 2006). However, SAG treatment failed to restore normal cerebellar long-term depression and working memory. Moreover, a key limitation of systemic targeting of the SHH pathways is the potential for such treatment to increase the risk of several types of human cancer (Taipale and Beachy, 2001; Jiang and Hui, 2008). An alternative strategy has been proposed based on use of a γ-secretase inhibitor to reduce overexpression of the SHH receptor PATCHED1 (PTCH1), which represses the SHH pathway. Overexpression of PTCH1 has been reported in Ts65Dn neural precursor cells (NPCs) (Trazzi et al., 2013, 2011) and is thought to result from the over-accumulation of the amyloid precursor protein intracellular domain (AICD), which is produced by cleavage of the APP precursor. The increase in AICD leads to overexpression of *PTCH1* in trisomic NPCs, impairing neurogenesis and neurite development (Trazzi et al., 2013, 2011). Using an inhibitor of APP γ-secretase, Giacomini et al. (2015) restored neuronal differentiation of Ts65Dn-derived NPCs and, with postnatal treatment, restored neurogenesis in the DG and the subventricular zone of Ts65Dn mice, while also normalising processing of APP. Thus, indirect targeting of the SHH pathway could form the basis of new therapeutic strategies to restore neurogenesis in trisomic brains.

An alternative is stem-cell-based therapy, a promising strategy for many diseases, including DS. Several studies have attempted to implant euploid neural stem cells into the brains of Ts65Dn mice and there is growing evidence that injected cells migrate to sites of damage where they provide neuroprotection. When NSCs were implanted in 12-month-old Ts65Dn mice, extrasomatic granules positive for expression of TAU and REELIN, associated with neuronal ageing, were reduced (Kern et al., 2011). When injected earlier, at P2, these cells induced a significant increase in the density of dentate granule cells and had a long-lasting positive effect on cognitive performance (learning) (Rachubinski et al., 2012a,b). Nevertheless, the stem-cell-based strategy has three limiting factors: (1) cells need to be injected directly into the brain for maximum efficacy; (2) the short-term effect is limited to the injection site and close vicinity; and (3) benefits are transitory and, when they persist, effects are probably linked to NSC-dependent neurotrophin production (Rachubinski et al., 2012b).

Among the other strategies that have been tested in DS mouse models is long-term peripheral administration of peptide 6 – an 11-mer corresponding to an active region of ciliary neurotrophic factor – which can enhance the pool of neural progenitor cells, and ameliorate learning and memory impairments in Ts65Dn mice (Blanchard et al., 2011). Three months of treatment with P7C3, an aminopropyl carbazole that enhances hippocampal neurogenesis, is sufficient to restore the neurogenic deficits observed in the Ts65Dn model (Latchney et al., 2015). Prenatal treatment with epigallocatechine gallate (EGCG), an inhibitor of DYRK1A, normalised some craniofacial phenotypes, including the increased cranial vault, in Ts65Dn mice (McElyea et al., 2016). Different protocols have demonstrated a beneficial impact of environmental enrichment – a widely used paradigm that increases sensory-motor stimulation – on learning, memory and motor activity in Ts65Dn mice (Llorens-Martín et al., 2010; Kida et al., 2013; Begenisic et al., 2015). The underlying molecular mechanisms responsible for this rescue are poorly understood, although it is thought that increased neurogenesis and synaptogenesis might be involved.

As highlighted above, the mTOR pathway has been implicated in DS. Treatment with the specific mTOR inhibitor rapamycin improved the spatial-memory performance of Ts1Cje mice and restored BDNF-dependent LTP in hippocampal slices (Andrade-Talavera et al., 2015). The same authors showed that deficits in synaptic plasticity (i.e. BDNF-LTP) and in the persistence of spatial memory were fully reversed using rapamycin in the Ts65Dn model (Andrade-Talavera et al., 2015), indicating that targeting mTOR hyperactivation may be a novel pharmacotherapeutical approach for DS. Consistent with this, administration of α-Tocopherol (vitamin E), also known to act upon the mTOR pathway, led to attenuation of cognitive impairments in the Ts65Dn model (Lockrow et al., 2009; Shichiri et al., 2011).

Different neurotransmission pathways have been shown to be altered in mouse models of DS, and these pathways have been targeted in attempts to improve cognition. In one example, Ts65Dn mice were treated with JZL 184, a selective inhibitor of monoacylglycerol lipase. The lipase degrades the most abundant endocannabinoid and its inactivation improves synaptic plasticity and memory in mouse (Pan et al., 2011). Thus, hippocampal LTP, long-term memory and locomotor activity were restored to a normal level in JZL-184-treated Ts65Dn mice; however, positive thigmotaxis (Box 1) and short-term memory defects remained unchanged (Lysenko et al., 2014).

Ts65Dn mice show perturbations of the excitatory/inhibitory balance towards an excess of GABA transmission; this imbalance may also explain cognitive dysfunction in human DS. The ability of antagonists of GABA receptors [including pentylenetetrazol (PTZ), RO4938581 and CGP55845] to normalise this balance has been tested in mouse models. These compounds rescued long-term and spatial memory, hippocampal LTP and the expression of several key molecular markers, including BDNF, GAD65/67 and VGAT (Fernandez et al., 2007; Rueda et al., 2008a; Braudeau et al., 2011a,b; Colas et al., 2013; Vidal et al., 2012; Kleschevnikov et al., 2004, 2012b; Martinez-Cue et al., 2013; Costa and Grybko, 2005). Similarly, the N-methyl-D-aspartate receptor antagonists memantine and MK-801 successfully rescued learning and improved memory, partly by normalising the levels of APP, vGlut1 and BDNF (Costa et al., 2008; Ahmed et al., 2015; Rueda et al., 2010; Lockrow et al., 2011; Hanson et al., 2013). A memantine clinical study is currently underway to assess the effects of this treatment on adolescents with DS (https://www.clinicaltrials.gov/).

Prenatal or early postnatal treatment with fluoxetine, which targets the serotoninergic system, rescued neurogenesis, long-term memory and synaptic plasticity in Ts65Dn mice (Guidi et al., 2014; Stagni et al., 2015). Adult treatment enhanced neurogenesis without improving learning/memory deficits at a high dose of fluoxetine (Heinen et al., 2012); however, an improvement in short-term memory was observed using a low dose (Begenisic et al., 2014). As seen with fluoxetine treatment, early administration of lithium, another antidepressant drug acting also upon the serotonin pathway, fully rescued short- and long-term memory, and LTP as well as neurogenesis in Ts65Dn mice (Contestabile et al., 2013).

Therapeutic targeting of key candidate genes implicated in DS has also been attempted. Partial display of DS phenotypes in mice harbouring a single trisomy of *Kcnj6* provides compelling evidence for a functional role of increased channel expression in some of the abnormal neurological phenotypes found in DS. However, treatment of Ts65Dn mice with ethosuximide (ETH), an inhibitor of KCNJ6, failed to rescue impairments of motor coordination or cognitive performance (MWM or FC) (Vidal et al., 2012). Given its putative role in DS, researchers have also explored the potential of *DYRK1A* as a therapeutic target. Studies involving inhibition of DYRK1A enzymatic activity using a safe and naturally available inhibitor (EGCG) demonstrated that learning/memory phenotypes could be rescued in different models, including in Ts65Dn mice (Xie et al., 2008; Guedj et al., 2009; De la Torre et al., 2014; Thomazeau et al., 2014), despite the fact that other genes are trisomic in this model.

As mentioned above, individuals with DS are at increased risk of developing AD during ageing, and the presence of three copies of APP is known to contribute to this phenomena. Thus, several therapies targeting the APP pathways have been tested. For example, the γ-secretase inhibitor DAPT was shown to improve memory and prevented neurodegeneration in Ts65Dn mice (Netzer et al., 2010). Anti-Aβ vaccine failed to rescue a normal level of Aβ40 and 42 but improved spatial learning of trisomic mice (Belichenko et al., 2016), suggesting that there is no direct relationship between the levels of Aβ and defects in spatial learning. In wild-type rodents, supplementing the maternal diet with additional choline (∼4.5× the amount in normal chow) enhances spatial memory and attention in the offspring, and exerts structural and functional changes in the septo-hippocampal cholinergic system (McCann et al., 2006): these results suggested that maternal choline supplementation (MCS) could enhance cognitive function and protect against BFCN degeneration. MCS given to Ts65Dn mice significantly improved spatial mapping and increased the number, density and size of medial septum BFCNs (Moon et al., 2010; Ash et al., 2014). MCS also seems to protect against hippocampal cholinergic projection system degeneration (Kelley et al., 2016).

Long-term exposure to environmental enrichment reduces Aβ oligomers and rescues spatial-memory abilities in 12-month-old trisomic mice (Sansevero et al., 2016). Estrogen treatment partially rescued working memory (T-maze test) and prevented neurodegeneration in aged Ts65Dn animals (11- to 17-months old) (Granholm et al., 2002, 2003). Consistent with a role for inflammatory processes in BFCN degeneration, minocycline treatment inhibits microglial activation, prevents progressive BFCN decline and markedly improves performance of Ts65Dn mice on a working and reference memory task (Hunter et al., 2004a). Partially reducing β-secretase 1 (BACE1) by deleting one *BACE1* allele blocked development of age-related endosome enlargement in the medial septal nucleus, cerebral cortex and hippocampus, and prevented loss of choline acetyltransferase (ChAT)-positive medial septal nucleus neurons (Jiang et al., 2016).

These potential therapeutic approaches have been tested ‘preclinically’ in DS mouse models (mostly in Ts65Dn mice) across different laboratories. Many different compounds and manipulations have produced beneficial effects. However, apart from EGCG and GABA-targeting compounds, the efficacy of specific strategies have not been validated in an independent study. Testing for interlaboratory reproducibility of the results, and reproducibility in more than one mouse model, will be crucial next steps in this exciting research area.

## Future directions for DS research

As highlighted here, DS mouse models are providing important insight into this complex disorder. Chromosome engineering technologies now enable us to address more refined questions, including, for example, on synergy and epistatic interaction between Hsa21 genes. The panel of new technologies provides the opportunity to broaden the range of animal models to include the rat or primates, although it must be emphasised that such models should only be developed where justified by the scientific question and there is clear added value compared to available models. A key limitation of DS mouse models is that most of the phenotypes have been described in a few genetic backgrounds, mostly pure inbred C57BL/6J mice, and for a few lines only in the F1, potentially reducing complex genetic interactions and their influence on the penetrance and expressivity of the phenotypes. It is widely known that the genetic background can impact on phenotypic outcome in mice; for example, in some backgrounds, homozygous knockout of epidermal growth factor receptor (EGFR) is lethal, whereas, in other backgrounds, the mutant is viable (Threadgill et al., 1995; Sanford et al., 2001). An impact on phenotype is also observed when modelling intellectual disabilities, autism spectrum disorders or psychiatric diseases (Sittig et al., 2016; Arbogast et al., 2016). Thus, addressing the interindividual phenotypic variability in DS will require strategies either to develop models in various genetic mixed backgrounds, e.g. with the F1 modelling approach proposed by Sittig et al. (2016), or to bring new genetic diversity using, for example, the Collaborative Cross lines (http://csbio.unc.edu/CCstatus/index.py) or using large cohorts derived in an outbred genetic background. In parallel, the use of large cohorts of ethnically and genetically diverse DS individuals will help to capture the variability in the human population.

The complex nature of DS creates the need for collaborative, multidiscliplinary research to understand its biology. One key step was the formation of the T21 Research Society (T21RS), a global initiative that provides freely available, curated and therefore up-to-date web resources, including validated models and protocols for behavioural and pharmacological treatment. The T21RS society is unique and aims to develop and coordinate action to generate new models to make them available and to propose standard operating protocols for behavioural investigation of DS. Moreover, mouse-based research into DS has resulted in the advancement of a few different treatments to human clinical trials. Promising results have been obtained in terms of improvement of cognitive function and the autonomy of individuals with DS (de la Torre et al., 2016, 2014), but more work is needed to better assess drug efficacy, the consequence of long-term treatment and the impact of treatment timing. In addition, most of the treatments were evaluated in a Ts65Dn mouse model, which only recapitulates the trisomy partially, lacking about half of the mouse genes that are homologous to those on Hsa21. The use of more complex mouse models, such as Dp1Yey and Dp1Tyb or those that combine trisomies for Mmu16, Mmu17 and Mmu10 homologous regions (Yu et al., 2010b), should be developed for reliable therapeutic assessment.

Models developed in other species, such as rat, are likely to be helpful to validate genotype-phenotype relationships and the outcome of preclinical treatments. Furthermore, we may have access to new avenues for therapeutic intervention. Firstly, we could adapt a treatment to the age of DS individuals, so aiming to counteract neurodevelopmental defects as early as possible, and decreasing the risk of comorbidities in the adult and during ageing. Before devising strategies for prenatal treatment, it is important to understand in depth the development of disomic versus trisomic human foetuses. New tools will also certainly contribute to new strategies. Notably, the use of CRISPR/Cas9 or CRISMERE tools could lead to therapeutic strategies for reducing the overdosage of a given gene or a genomic region in trisomic individuals by inactivating one of the three alleles, for example, in transplantable stem cells or induced pluripotent stem cells. Finally, DS models will continue to be improved in the next few years. As noted above, most drug candidates to date have been tested in the Ts65Dn model, which carries only ∼90 of the Hsa21 homologous genes and includes ∼60 additional genes that are not orthologous to Hsa21. Thus, we must consider more complete models to evaluate therapeutic options even though these may be difficult to breed at present (Belichenko et al., 2015). An intriguing alternative is provided by the transchromosomic strategy that brings a complete set of Hsa21 genes into the mouse genome. However, this approach has some pitfalls: (1) human proteins or non-coding RNAs may not be able to interact with the same efficiency and efficacy with their mouse partners; (2) the regulation of human sequences may differ from the mouse genes, leading to unexpected domains of expression; and (3) the human allelic repertoire will be fixed in each strain. Despite the limitations, the progress to date has been promising and there is little doubt that mouse-based DS research will be important for translation into therapies for DS in the future.
